# Comment on Du et al. Gender Differences in the Relationships between Perceived Stress, Eating Behaviors, Sleep, Dietary Risk, and Body Mass Index. *Nutrients* 2022, *14*, 1045

**DOI:** 10.3390/nu14193881

**Published:** 2022-09-20

**Authors:** Wan-Chin Kuo, Jennifer M. Stevens, Elliot A. Tebbe

**Affiliations:** School of Nursing, University of Wisconsin-Madison, Madison, WI 53705, USA

We read Du et al. [1] with great interest, which examined the moderated mediating effects of sleep and emotional eating in the association of perceived stress with the body mass index (BMI). This study provides a helpful opportunity for discussion of the principles of mediation analysis in health research.

Conventional approaches to mediation analysis have followed recommendations by Baron and Kenny (B&K) [2], who argue that to quantify the extent to which a variable (M) mediates the relationship between an independent variable (IV) and dependent variable (DV), one must first demonstrate a significant total effect (IV→DV), significant paths in IV→M and M→DV, and a significant indirect effect through M (IV→M→DV). However, MacKinnon and others recommend estimating confidence limits by using critical values for the asymmetric distribution of the product or resampling methods (e.g., bootstrapping), because the indirect effect is the product of two coefficients (IV→M and M→DV) and the product may not follow a normal distribution [3,4,5]. Furthermore, simulation examples have found mediation when B&K’s criteria are not satisfied, demonstrating that it is possible to have statistically significant indirect effects in the absence of a total effect, especially when several mediating paths have opposite signs that cancel each other out [3,6].

In this study of 1392 students, Du et al. used bias-corrected bootstrap confidence limits for mediation analysis. They identified a small indirect effect where emotional eating mediated the association between perceived stress and the BMI in female students (95% CI = 0.01, 0.04). However, had Du et al. followed B&K’s approach, it is likely that they would not have found a significant indirect effect; simulation studies have demonstrated a minimum sample size of 20,886 for B&K’s approach to detect small mediation effects in a completely mediated model with a power of 0.8 [3]. Furthermore, given the weak bivariate correlation between perceived stress and BMI (r = 0.055, *p* < 0.05), regressing BMI on perceived stress with covariates might have led to a nonsignificant IV→DV, precluding mediation findings through B&K’s approach.

As a seminal article, B&K’s approach should be introduced, balanced with its limitations, to all students as they learn mediation analysis: It is theoretically easy to understand, can be examined using regression models, and has conservative type I error rates [7]. Loeys et al. recommend careful consideration at the design stage regarding common factors that might influence mediators and outcomes and conducting sensitivity analysis to mitigate the impact of confounding effects [7]. For example, consider whether sleep quality/duration introduces spurious effects within the mediation model (Figure 1A) prior to conducting moderated mediation analyses (Figure 1B). We believe that Du et al.’s study provides an example for researchers to see how other approaches of mediation analysis might lead to different conclusions and that sensitivity analysis might be needed when confounding effects exist.

## Figures and Tables

**Figure 1:**
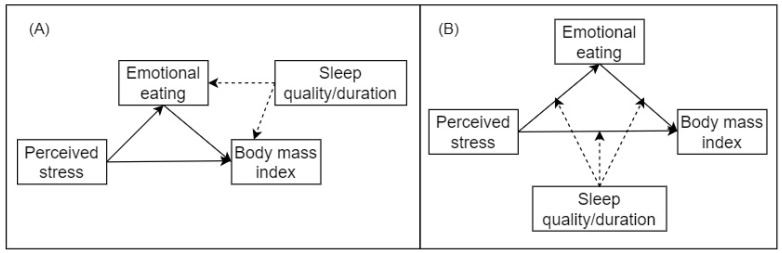
Conceptualization of the mediation model during the design stage: (**A**) potential confounding variables (sleep quality and sleep duration) introducing spurious effects in the mediation model; (**B**) the moderated mediation model proposed in Du et al.’s study.
